# Differential Associations Between Distinct Components of Cognitive and Physical Function in Middle-Aged and Older Adults

**DOI:** 10.3390/brainsci16010040

**Published:** 2025-12-27

**Authors:** David Facal, Eduardo Picón, Helena M. Blumen, Cristina Lojo-Seoane, Ana Nieto-Vieites, Yaakov Stern, Arturo X. Pereiro

**Affiliations:** 1 Department of Developmental and Educational Psychology, IDIS, University of Santiago de Compostela, 15782 Santiago de Compostela, Spain; cristina.lojo@usc.es (C.L.-S.); ananieto.vieites@usc.es (A.N.-V.); arturoxose.pereiro@usc.es (A.X.P.); 2 Institute of Psychology (IPsiUS), University of Santiago de Compostela, 15782 Santiago de Compostela, Spain; 3 Department of Methodology of Behavioral Sciences, University of Santiago de Compostela, 15782 Santiago de Compostela, Spain; eduardo.picon@usc.es; 4 Department of Neurology, Stony Brook University, Stony Brook, NY 11794, USA; helena.blumen@stonybrookmedicine.edu; 5 Cognitive Neuroscience Division, Department of Neurology, Columbia University Vagelos College of Physicians and Surgeons, New York, NY 10032, USA; ys11@cumc.columbia.edu

**Keywords:** verbal learning, executive function, mobility, Timed-Up and Go, grip strength, non-parametric analysis

## Abstract

**Background**: Cognitive and physical functions share certain age-related patterns of change, including slowed processing speed and movement. Both functions are multifaceted, and the association between them can be affected by the type of measurement considered. This study examined one-to-one relationships between cognitive and physical functions, using data from the Compostela Aging Study. **Methods**: A total of 267 middle-aged and older individuals without cognitive impairment were included in the study (mean age 65.57, 75.7% women). The relationship between cognitive and physical performance was examined using Spearman’s rho, adjusted for age and sex. **Results**: Standing up, sitting down and total times in the Timed-Up and Go test were significantly correlated with performance on the Trail-Making and phonological fluency tests. Turning time in the Timed-Up and Go test and self-reported physical activity were correlated with performance on the Spanish version of the California Verbal Learning Test. Grip strength was correlated with performance on the Counting Span task. **Conclusions**: This study adds evidence to the one-to-one relationship between cognitive and physical function in a subclinical cohort of middle-aged and older adults.

## 1. Introduction

Physical functioning is the ability to carry out the physical activities that are necessary for daily living [1]. Cognitive and physical functions share age-related patterns of change, including slowed processing speed and movement in aging and shared neural substrates [2,3,4]. The use of non-cognitive markers in research on cognitive aging has been common since Baltes and Linderberger suggested that the link between cognition and motor–sensory function reflects a decline in central nervous system functioning [5]. Cognition is important for locomotion, and functional magnetic resonance imaging (fMRI) imagined-gait protocols suggest activation of cerebellar, precuneus, supplementary motor and prefrontal regions in older adults [6]. Positive pattern weights show that activation of the bilateral cerebellum, bilateral precuneus and several prefrontal cortex regions increases significantly with cognitive load in the imagined-gait task. By contrast, negative pattern weights show decreased activation in occipital, middle temporal, medial frontal and cingulate regions. In another study of gray matter volume covariance networks in older adults, normal pace walking was associated with the structure of the supplementary motor area, the precuneus cortex and the middle frontal gyrus, while dual-task costs in a walking-while-talking test were associated with more extended medial prefrontal, cingulate and thalamic regions [7]. The relationship between cognitive function and physical function increases in the presence of even subtle age-related decline [8]. Gait decline has also been identified as an early and reliable predictor of cognitive impairment [2,9,10,11,12]. The opposite pattern has also been observed, with impaired cognitive control of gait in MCI and early dementia [13,14,15]. Assessing gait parameters such as step variability is useful for the early detection of cognitive impairment, as it increases explanatory and predictive capacity [16].

Cognitive and physical functions are multifaceted, and the association between them can be affected by the specific functions considered and by the type of measurements considered [17]. Various studies show the importance of the variables selected when studying the relationship between cognitive and physical functions during the aging process. For example, Holtzer et al. [3] found that the cognitive factor that most strongly contributed to predicting gait velocity, in both single and dual-task walking conditions, in a sample of cognitively normal older adults, was executive speed/attention, followed by memory. Sprague et al. [1] found that grip strength was a significant predictor of memory, attention and reasoning performance in the oldest-old group. In addition, a significant curvilinear relationship between a turning task and attention was observed, indicating that turning ability was a stronger predictor of attentional performance for younger and older people than for middle-aged adults. Using single- and dual-task gait and the Timed-up and Go (TUG) test as mobility measures, Sunderaraman et al. [4] observed significant associations between executive functions and turn duration, range of sit-to-stand, dual-task stride variability and dual-task step symmetry, between language measures and dual-task stride time variability, dual-task stride regularity and dual-task pace, and between processing speed and dual-task step regularity and dual-task stride regularity. No significant relationship between memory and any of the mobility measures was found in the study. Tripathi et al. [7] found that a gray matter volume covariance network associated with normal pace walking varied with processing speed and executive function, whereas gray matter volume covariance networks of walking-while-talking dual costs varied with episodic memory. Finally, Verghese et al. found that changes in rhythm (factor loadings including cadence and timing measures such as swing time and stance time) and pace (including gait speed, stride length and double support time) are associated with decline in memory and in executive function, respectively [18].

Altogether, previous findings indicate that the nature of the cognitive–physical relationship varies depending on the specific domains and measures used and may be further shaped by demographic factors such as age and sex. Building on this evidence, the present study aimed to examine one-to-one relationships between cognitive and physical functions in the Compostela Aging Study (CompAS) sample, specific patterns of relationships between different cognitive functions and different physical functions, illustrating shared factors among specific cognitive and physical functions in supporting global motor behavior.

## 2. Materials and Methods

### 2.1. Participants

Participants were selected from the 2nd and 3rd cohorts of the CompAS, an ongoing longitudinal study that assesses and follows middle-aged and older adults with self-reported cognitive concerns. Participants in the CompAS are recruited from primary care centers in the Santiago de Compostela health area. To avoid the complex procedures involved in handling missing data, only participants for whom complete cognitive and physical data were available were considered. Of the 391 participants who had completed cognitive and physical assessments, 267 who were not diagnosed with MCI were selected for inclusion in the present study (mean age = 65.57, S.D. = 8.25, min = 50, max = 89; 202 women, 75.7%). Thus, the CompAS participants selected did not present with objective cognitive impairment in the neuropsychological examination, according to normative values for age and educational level. The selection was made considering the objective of examining the one-to-one relationships between cognitive and physical function across middle age and old adulthood and also to avoid interpretation of the results being complicated by the presence of participants with neuropathology. In addition, none of the participants had a previous diagnosis of dementia, psychiatric or neurological disorders, serious illness, deafness or blindness, were receiving chemotherapy or had history of alcohol or other substance abuse. Participants had completed on average 13.54 years of education (S.D. = 5.62, range 2–28 years), reported having an average number of 8.88 close friends or family members in their social network (S.D. = 8.89, range 1–100) and had an average Charlson Comorbidity Index score of 0.27 (S.D. = 0.57, range 0–3).

Two age categories were established, to enable age-related comparisons: middle-aged individuals, up to 64 years old, and older adults, aged 65 and above. The middle-aged group consisted of 122 participants (103 women, 84.4%), of mean age 58.22 years (SD = 3.73, range 51–64 years), with an average of 15.64 years of education (SD = 4.96, range 5–26 years), a reported average of 7.80 close friends or family members in their social network (S.D. = 7.94, range 1–76) and an average Charlson Comorbidity Index score of 0.16 (S.D. = 0.43, range 0–2). The older adult group consisted of 145 participants (99 women, 68.3%), of mean age 71.75 years (S.D. = 5.45, range 65–89 years), with an average of 11.78 years of education (S.D. = 5.55, range 2–28 years), a reported average of 9.79 close friends or family members in their social network (S.D. = 9.55, range 1–100) and an average Charlson Comorbidity Index score of 0.36 (S.D. = 0.65, range 0–3).

### 2.2. Instruments

#### 2.2.1. Cognitive Functions

Cognitive assessment included measures of memory, assessed with the Spanish version of the California Verbal Learning Test (CVLT); language, assessed with the Spanish version of the Boston Naming Test (BNT), 3rd edition, semantic and phonological fluency tasks, and also executive function, assessed with the Trail Making Test form A (TMT-A) and form B (TMT-B), and a counting span task.

In the Spanish version of the CVLT, a list of 16 words simulating a shopping list is orally presented to participants, who are required to recall the words immediately and after short and long delays [19]. This study includes total immediate recall after each of the 5 presentations of the word list, a short delay free recall (SDFR) score, and a long delay free recall (LDFR) score.

The BNT is the most widely used naming test, requiring individuals to name line drawings of 60 objects graded by naming difficulty [20,21]. The test was applied according to the reference manual, and the total score for the 60 items was recorded.

Fluency tasks were applied considering the procedures established within the Spanish Consortium for Ageing Normative Data (SCAND) for semantic and phonological fluency. TMT-A and TMT-B [22] were used to measure visual-perceptual processing and working memory, respectively. TMT-A requires participants to connect a series of encircled numbers in ascending order as quickly and accurately as possible. TMT-B presents a more complex sequencing condition in which participants alternate between numbers and letters in ascending order (e.g., 1–A–2–B–3–C). For execution of both TMT-A and TMT-B, the examiner instructed participants to proceed as rapidly as possible without lifting the pencil from the paper and to correct any errors before continuing. The outcome measure is the time in seconds required to complete each form, with longer completion times indicating slower processing speed (TMT-A) or reduced executive functioning (TMT-B).

Finally, the Counting Span task is a working memory span task in which a random number of target items (dark blue circles) are presented on slides in which non-target items that share a feature with the targets (light blue circles and dark blue squares) are presented at the same time [23]. Participants were asked to count aloud and recall the number of dark blue circles shown in each slide. The total number of items recalled was recorded in this study.

#### 2.2.2. Physical Function

Physical assessment included a TUG test, grip strength, and a self-reported physical activity test. The TUG is a modification of the Get Up and Go test in which the score is the time it takes the person to get up from a chair, walk 3 m, turn around, walk 3 m back to the chair, and sit down. A wireless inertial sensor (Wiva^®^, Loran Engineering, Bologna, Italy) was used to record the performance in the TUG. The sensor, which includes an accelerometer, a triaxial magnetometer and a gyroscope, provides reliable partial times of the main phases of the TUG test [24,25]. The sensor, of size 35 × 37 × 15 mm, was attached to the body at the level of the lumbar region, and a Bluetooth connection was used for data transmission. It has shown high reliability for spatiotemporal gait parameters at comfortable walking speeds [26]. Two evaluators were trained by a team member of the company on how to affix the sensor to participants and how to operate the device software.

TUG transitions and turning reflect additional and independent mobility domains in the context of age-related mobility changes [4,27]. Therefore, the time spent to perform the entire task, and also the time spent to the transition from sitting to standing, the time to turn around the cone, and the time to transition from standing to sitting were recorded in this study.

Grip strength was recorded, in kilograms, using an electronic hand dynamometer (CAMRY EH101 dynamometer, General ASDE, Valencia, Spain). Three measurements were made using the participant’s dominant hand, and the mean score was calculated [28].

Self-reported physical activity was measured using the short Spanish version of the Minnesota leisure time Physical Activity Questionnaire (VREM) [29]. Energy consumption (METS-min/14 days) was estimated from the results of this test.

### 2.3. Procedure

CompAS is an ongoing cohort study aimed at identifying early markers of cognitive impairment in middle-aged and older adults in Santiago de Compostela and Vigo (Galicia, NW Spain). All participants were referred to the study by health professionals from public primary health care centers after reporting subjective cognitive complaints. Trained psychologists carried out a comprehensive assessment of the participants in the primary health care centers, including a first session aimed at screening and diagnosis, and a second session that included complementary neuropsychological and affective assessment. Most of the tests and tasks included in this study were applied in the first session, except the Counting Span task, which was applied in the second session.

The procedure was approved by the Galician Clinical Research Ethics Committee (Ref. 2022/116). All procedures were performed in accordance with the ethical standards of the Spanish and European legislations and with the 1964 Helsinki Declaration and its later amendments. Written informed consent was obtained from all individual participants included in the study.

### 2.4. Data Analysis

Differences between middle-aged and older groups and between female and male groups were examined using the non-parametric Mann–Whitney U test. The matrix of correlations between the cognitive and physical variables was examined using Spearman rho correlation coefficients, adjusted for age (in years) and sex. Following a detailed data quality review, we decided not to carry out non-linear transformations, in order to preserve the natural variability of the data. Significant correlations (*p* < 0.050) are graphically represented as scatter plots. The age- and sex-adjusted partial correlations used for these graphs were extracted from the unstandardized residuals of linear regression analyses. All statistical tests were conducted using SPSS 29.0.2.0.

## 3. Results

Descriptive statistics for the whole sample and for the different groups categorized by age and sex are presented in Table 1, Table 2 and Table 3 respectively, and the results of non-parametric tests for differences between the different groups are presented in Table 4. Significant differences between middle-aged and older adults were observed for all cognitive measures and total, standing and sitting time in the TUG. No differences were found for turning time in the TUG, grip strength or self-reported physical activity.

**Figure 1:**
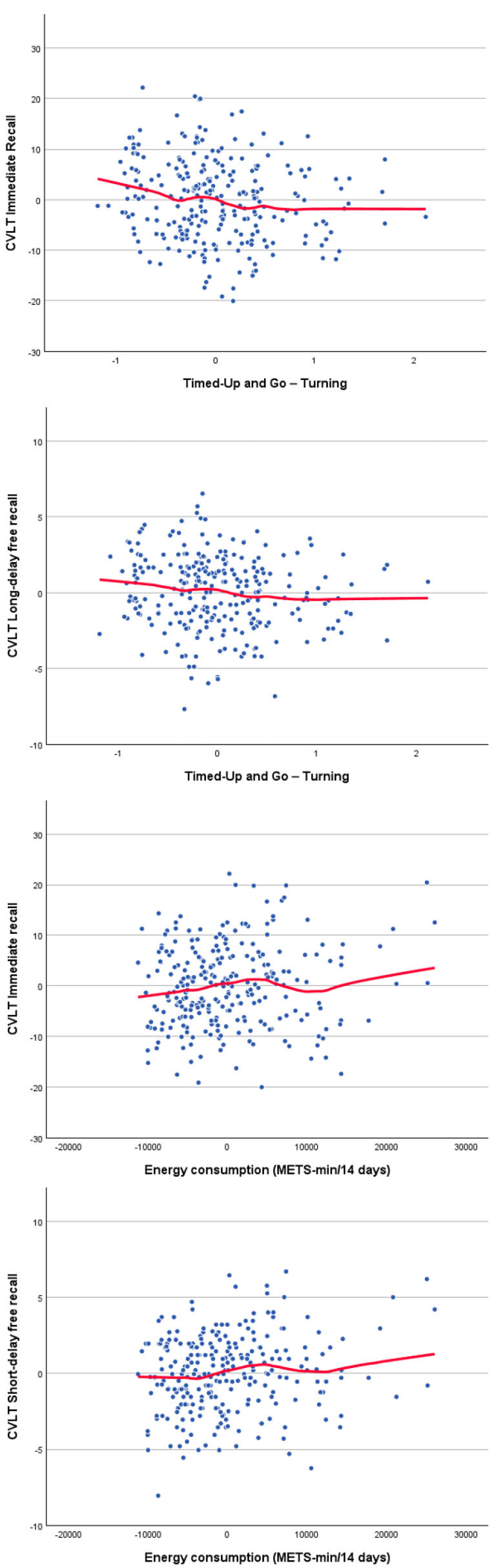
Scatter plot for significant age- and sex-adjusted partial correlations between the California Verbal Learning Test (CVLT) performance residuals and both the Timed Up and Go (TUG) turning time and the self-reported physical activity residuals. The red lines show the loess fitting curves.

**Figure 2:**
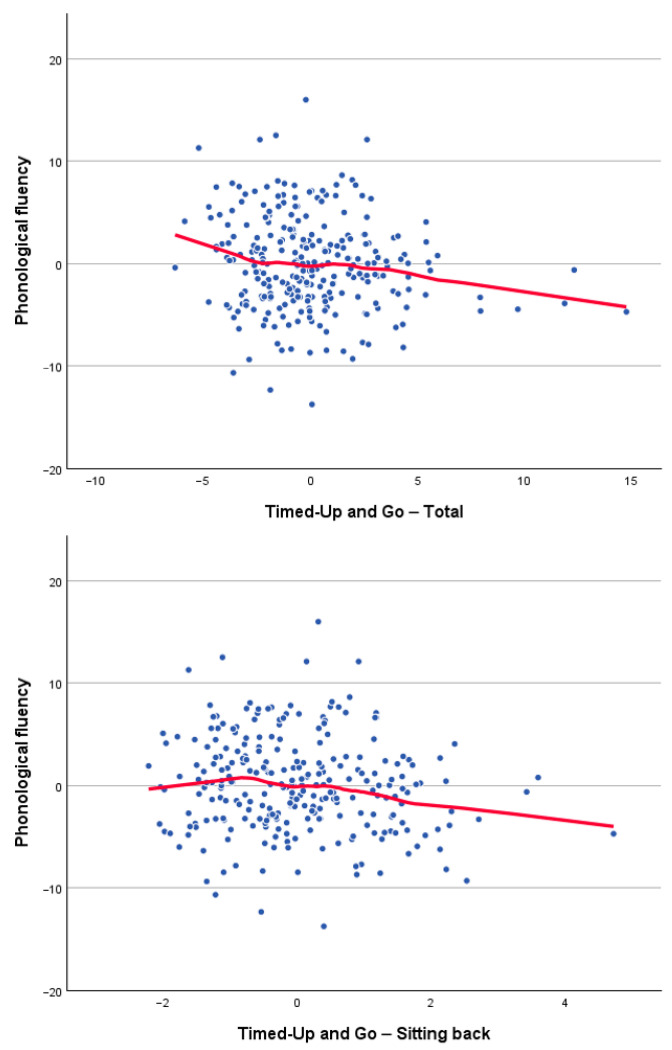
Scatter plot for significant age- and sex-adjusted partial correlations between phonological fluency residuals and both Timed Up and Go (TUG) total time and sitting back down time residuals. The red lines show the loess fitting curves.

**Figure 3:**
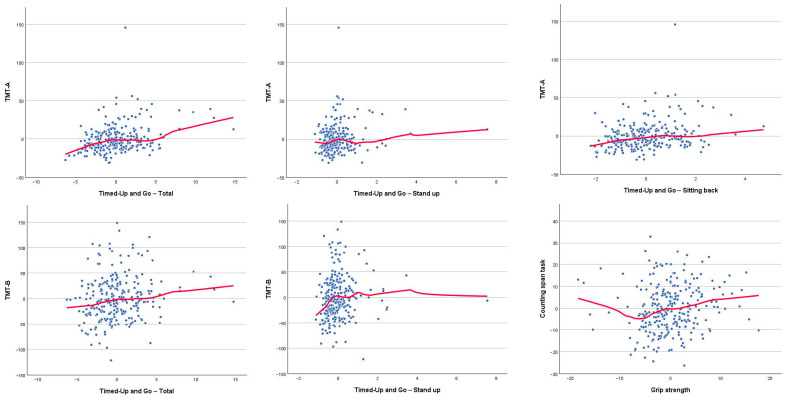
Scatter plot for significant age- and sex-adjusted partial correlations between Trail Making Test (TMT) part A residuals and Timed Up and Go (TUG) total time, standing up time and sitting back down time residuals; between TMT part B residuals and TUG total time and standing up time residuals; and between Counting Span Task residuals and grip strength residuals. The red lines show the loess fitting curves.

Correlations between cognitive and physical functioning, controlling for age and gender, are presented in Table 4. The significant associations between physical function and CVLT scores (Figure 1) revealed small but consistent relationships with turning performance during the TUG and with self-reported physical activity. Significant correlations between physical function and phonological fluency (Figure 2) indicate that individuals with higher phonological fluency tend to complete the TUG, particularly the sitting-back down stage, more rapidly. Furthermore, significant associations between TUG components and TMT performance (Figure 3) indicate that poorer processing speed and executive functioning are related to slower motor performance. Finally, the significant correlation between counting-span performance and grip strength (Figure 3) suggests a link between greater working-memory capacity and greater physical strength. Together, these findings show small but consistent links between cognitive and physical performance after adjusting for age and sex. Thus, the observed relationships reflect unique associations between cognition and motor performance, rather than simply the effects of being older and/or male/female. Measures of processing speed and executive functioning showed the clearest pattern, with TMT performance being significantly correlated with multiple components of TUG. Individuals who performed the TMT more slowly tended to spend more time completing the TUG, suggesting that reductions in attentional control and cognitive flexibility are linked to slower motor execution. Phonological fluency also displayed meaningful correlations with TUG performance, in contrast to language measures with lower executive demands, such as semantic fluency and naming, which were not significantly related to physical variables. Together, these results reinforce the view that executive-demanding tasks involving rapid information processing, controlled retrieval and planning share common underlying mechanisms with complex motor activities.

## 4. Discussion

Group comparisons in a sample of middle-aged and older adults with cognitive memory complaints, recruited from primary care centers, revealed a well-known, broad pattern of age-related decline in cognitive and motor functioning. Older adults performed significantly less well than middle-aged adults across all cognitive domains, highlighting the pervasive impact of aging on higher-order cognitive abilities. Motor performance showed a similar age-related pattern, with older adults requiring more time to complete most components of the TUG test, suggesting reduced mobility and postural control, although turning time and grip strength were preserved. By contrast, gender differences were limited: women outperformed men in verbal learning and verbal memory, consistent with established memory advantages [30], while men exhibited substantially greater grip strength, reflecting known physiological differences. However, no gender effects emerged in other cognitive tests, mobility measures or self-reported physical activity.

The study aimed to examine the one-to-one relationships between cognitive and physical functions and considering the influence of age and sex. After controlling for the effects of age and sex, we found significant correlations between (a) verbal learning and measures of mobility and self-reported physical activity, (b) phonological fluency and measures of mobility, and (c) executive function and measures of mobility and strength. Thus, this study adds evidence to the one-to-one relationship between cognitive and physical function in a subclinical sample. This is an important contribution as these relationships have been found to vary depending on the specific cohort studied, and convergent evidence is still needed [1,2,3,4].

Our results support previous associations between attention-executive functions and specific aspects of mobility. The strongest associations found were between TUG and TMT scores, specifically between time taken to complete TMT-form A and both TUG total time and sitting back down time, and between time taken to complete TMT-form B and TUG stand-up time. TUG performance is complex and requires considerable executive processing, involving cognitive resources for moving the body forward, accelerating in the vertical plane while standing, initiating stepping, sequencing deceleration, and preparation for turning to return to the sitting position. Although few studies have differentiated between the time taken to perform different subtasks, it has also been hypothesized that a long duration between subtasks will be associated with poorer cognitive function [28]. Sunderaraman et al. [4] reported significant associations between executive function and two TUG measures-duration of turning and amplitude of transition from sitting to standing. In a systematic review, Morris et al. [10] found a strong association between pace and attention–executive function. These researchers hypothesized a relationship between this association and the increased number of white matter lesions in older adults, with age-related degeneration in white matter eliciting decline in both pace and executive attention, predominantly due to cholinergic burden. Tripathi et al. [7] reported significant patterns of gray matter volume associated with pace, including in control areas such as bilateral supplementary motor and cerebellar areas, as well as in regions involved in multi-sensory information processing and in motor (automatic) pathway areas. Older adults appear to require more brain activity for automatic performance at the same level as young subjects [31,32]. De-automatization of processes in older adults would explain the growing importance of executive processes in performing automatic movements such as walking.

Contrary to the findings of Sunderaraman et al. [4], who reported relatively strong relationships between language and gait measures in a language-based dual task condition, we did not find any significant associations between the language measures (BNT, semantic fluency) and physical performance. The only significant relationships were between both TUG total time and TUG sitting back down time and phonological fluency. The role of language processing and executive components of verbal fluency remains controversial [33,34]. However, because phonological fluency rarely occurs naturally in daily life and requires active inhibition of large numbers of competing words, it is often more closely associated with executive control mechanisms than semantic fluency.

Turning duration has previously been related to executive function and processing speed [4]; however, in the present study it was most strongly related to verbal learning memory. Most studies on the hippocampus stress non-spatial memory functions such as those related to verbal learning tests, although some researchers also indicate a role for the hippocampus in spatial learning and spatial memory. Patients with bilateral vestibular loss developed atrophy of the hippocampus and showed significant spatial memory and navigation deficits in a virtual maze test, in the absence of general memory deficits [35]. Impairment on video-recorded real-world topographical orientation tasks were found in patients with unilateral left and right temporal lobe surgery relative to the normal control group [36]. Studies investigating the large hippocampal volumes of London taxi drivers also support the relationship between hippocampus and spatial representations of the environment in adults with normative development [37,38]. In accordance with this view, the integrity of the hippocampus required for optimal performance in the verbal learning test will also be necessary to integrate visuospatial clues involved in turning within the body movement required for the TUG test. Likewise, verbal episodic memory performance is also related to neural pathways supporting dual tasking, namely the medial pre-frontal cortex [7], which will be of greater relevance to more complex aspects of mobility-related tasks such as turning acceleration in the TUG test.

A relationship between working memory and grip strength was also observed in the present study. Beyond the complex integration and coordination of multiple body systems (sensory, motor, cognitive), grip strength requires relatively simple hand grasping performance, which is probably more directly related to the availability of processing resources and cognitive status. Potential mechanisms underlying the relationship between grip strength and cognition in healthy adults include the central nervous system functioning and changes in white matter integrity [39]. Sprague et al. [1] found that associations between grip strength and all cognitive domains (memory, processing speed/attention, reasoning) increased significantly with age. In a meta-analysis of the relationships between physical and cognitive functioning in longitudinal studies, Clouston et al. [15] found that change in grip strength was more strongly correlated with cognitive status, while other changes in physical functioning, such as changes in walking speed, were more closely related to change in fluid cognition. Future longitudinal studies should explore whether grip strength is a good measure for predicting diagnostic transitions linked to general cognitive status and/or for predicting the availability of processing resources in more specific tasks such as working memory span tasks.

Our findings are consistent with previous research showing that cognitive performance (especially executive function, attention and verbal learning) is linked to specific aspects of mobility and physical function. In line with earlier studies, we observed that measures related to TUG performance were most strongly associated with executive processes, supporting the view that gait and postural transitions recruit higher-order cognitive resources in aging populations. The findings thus highlight the potential for including mobility tests in early cognitive assessment protocols. Tools or tests that can identify subtle age- or disease-related changes in gait could be useful for improving cognitive health [40]. The findings also confirm that, as well as for physical fitness and functional improvement, regular physical exercise could be promoted as a cognitive health strategy, given the relationship with memory and learning capacities [41].

As in previous studies exploring one-to-one relationships between cognitive and physical function in aging cohorts [4], the main limitation of the present study is its exploratory and cross-sectional nature. Longitudinal studies are required for a better understanding of possible mediators associated with cognitive reserve and brain reserve in cognitive-motor correlations that can shed light on possible interventions. Furthermore, as different combinations of cognitive functions seem to operate to regulate specific physical domains, different connectivity paths must be established. In this study, we observed relationships between executive and memory functions and specific aspects of physical function, notably between executive function and TUG total time, standing up time and sitting back down time, between verbal learning memory and TUG turning time, and between working memory span and grip strength. However, these relationships are exploratory and must be confirmed by longitudinal studies and by establishing structural and/or functional brain correlates to support these relationships and make them more specific. Following the study aim, we only selected those CompAS participants who did not show any objective cognitive impairment. We believe that excluding participants with MCI provided a clearer perspective on the one-to-one relationships between different measures of cognitive and physical functioning. However, future studies, particularly more methodologically complex studies and/or studies with biomarkers, should also consider including participants with MCI. Finally, we only controlled for age and sex. More complex and comprehensive models should also include education and other relevant demographic or health factors that influence the relationship between cognitive and motor function [2].

## 5. Conclusions

This study shows an age-related decline in both cognitive and motor functioning in middle-aged and older adults with subjective memory complaints, alongside domain-specific gender differences. Beyond these differences, the findings provide convergent evidence for specific one-to-one associations between cognitive domains—particularly executive function, attention, verbal learning, and working memory—and distinct aspects of mobility, independent of age and sex. Measures derived from the TUG were most consistently linked to executive processes, underscoring the cognitive demands of gait and postural transitions in aging, while associations between verbal learning and turning time, and between working memory and grip strength, suggest partially distinct neural and functional pathways underlying different cognitive–motor relationships. Although exploratory and cross-sectional, these results support the notion that subtle changes in mobility may serve as early indicators of cognitive vulnerability and reinforce the potential value of incorporating mobility assessments into early cognitive screening. They also highlight physical activity as a promising strategy for supporting brain health. Future longitudinal studies are needed to confirm the proposed relationships.

## Figures and Tables

**Table 1:** Descriptive statistics (means, standard deviations and range) for the whole sample.

	Mean	Standard Deviation	Range
CVLT total immediate recall	54.15	10.37	25–76
CVLT short-delay free recall	11.40	3.33	2–16
CVLT long-delay free recall	12.08	3.05	2–16
Boston Naming Test total score	54.05	5.77	33–60
Semantic fluency score	20.78	6.16	6–37
Phonological fluency score	15.59	5.20	1–32
Trail making test—form A	45.22	20.82	15–180
Trail making test—form B	107.27	55.88	39–300
Counting span task—total items recalled	35.43	12.07	12–62
Timed-Up and Go—total time	10.89	3.22	5.56–26.95
Timed-Up and Go—standing up time	1.70	0.82	0.66–9.26
Timed-Up and Go—turning time	1.42	0.57	0.35–3.50
Timed-Up and Go—sitting back down time	2.01	1.19	0.14–6.72
Grip strength (Kg)	24.95	8.35	4.20–54.40
Self-reported physical activity (METS-min/14 days)	12,016.67	7031.16	880.11–38,453.14

**Table 2:** Means, standard deviations (between brackets) and age-group comparisons between middle aged and older adults.

	Age Group		
	Middle Aged	Older	Mann–Whitney U
CVLT total immediate recall	59.33 (8.01)	49.79 (10.15)	4258.00 **
CVLT short-delay free recall	13.21 (2.36)	9.88 (3.26)	3658.50 **
CVLT long-delay free recall	13.55 (2.07)	10.85 (3.20)	4370.50 **
Boston Naming Test total score	56.69 (3.43)	51.83 (6.38)	4424.00 **
Semantic fluency score	24.24 (5.43)	17.88 (5.19)	3563.50 **
Phonological fluency score	17.93 (4.97)	13.61 (4.54)	4611.50 **
Trail making test—form A	36.75 (18.09)	52.35 (20.33)	14,007.50 **
Trail making test—form B	76.48 (29.74)	133.17 (59.56)	14,363.50 **
Counting span task—total items recalled	40.36 (11.42)	31.29 (11.03)	4780.00 **
Timed-Up and Go—total time	10.07 (2.65)	11.58 (3.49)	11,189.00 **
Timed-Up and Go—standing up time	1.54 (0.66)	1.82 (0.91)	11,158.00 **
Timed-Up and Go—turning time	1.38 (0.52)	1.46 (0.62)	9215.50
Timed-Up and Go—sitting back down time	1.80 (1.12)	2.19 (1.21)	10,421.00 *
Grip strength (Kg)	25.88 (8.69)	24.17 (8.01)	7730.00
Self-reported physical activity (METS-min/14 days)	11,510.61 (6840.57)	12,442.45 (7183.49)	954,750

** *p* < 0.001; * *p* < 0.01.

**Table 3:** Means, standard deviations (between brackets) and age-group comparisons between females and males.

	Sex		
	Female	Male	Mann–Whitney U
CVLT total immediate recall	55.75 (9.72)	49.17 (10.80)	4204.50 **
CVLT short-delay free recall	11.90 (3.04)	9.85 (3.70)	4426.00 **
CVLT long-delay free recall	12.52 (2.75)	10.72 (3.54)	4628.00 **
Boston Naming Test total score	53.96 (5.85)	54.34 (5.55)	6676.00
Semantic fluency score	20.91 (6.33)	20.41 (5.67)	6298.00
Phonological fluency score	15.82 (5.16)	14.86 (5.29)	5995.00
Trail making test—form A	45.86 (21.24)	43.25 (19.46)	6032.00
Trail making test—form B	106.67 (54.68)	109.14 (59.86)	6684.00
Counting span task—total items recalled	35.73 (11.89)	34.52 (12.67)	6250.50
Timed-Up and Go—total time	10.74 (3.32)	11.33 (2.86)	7562.00
Timed-Up and Go—standing up time	1.70 (0.88)	1.68 (0.59)	6928.50
Timed-Up and Go—turning time	1.41 (0.56)	1.47 (0.62)	7026.50
Timed-Up and Go—sitting back down time	1.98 (1.20)	2.12 (1.13)	7231.00
Grip strength (Kg)	21.63 (5.39)	35.28 (7.47)	12,408.50 **
Self-reported physical activity (METS-min/14 days)	12,351.07 (7022.44)	10,977.45 (7010.32)	5768.00

** *p* < 0.001.

**Table 4:** Spearman RHO correlations between cognitive and motor variables adjusted for sex and age.

	Timed-Up and Go—Total Time	Timed-Up and Go—Stand Up Time	Timed-Up and Go—Turning Time	Timed-Up and Go—Siting Back Down Time	Grip Strength	Self-Reported Physical Activity
CVLT total immediate recall	−0.05	−0.06	−0.15 *	−0.02	0.09	0.13 *
CVLT short-delay free recall	0.03	0.06	−0.10	0.07	0.04	0.14 *
CVLT long-delay free recall	0.07	0.10	−0.13 *	0.09	0.07	0.12
Boston Naming Test total score	−0.01	−0.04	−0.11	−0.01	0.09	−0.02
Semantic fluency score	−0.01	−0.03	−0.07	−0.01	−0.03	0.03
Phonological fluency score	−0.12 *	−0.12	−0.03	−0.12 *	0.04	0.07
Trail making test—form A	0.22 **	0.15 *	0.10	0.20 **	−0.07	−0.05
Trail making test—form B	0.12 *	0.16 **	0.10	0.09	−0.10	−0.05
Counting span task—total items recalled	−0.09	−0.07	−0.07	−0.12	0.14 *	0.04

** *p* < 0.01; * *p* < 0.05.

## Data Availability

The data that support the findings of this study are available on request from the corresponding author due to privacy issues.

## References

[1] Sprague B.N., Phillips C.B., Ross L.A. (2019). Age-varying relationships between physical function and cognition in older adulthood. J. Gerontol. Ser. B.

[2] Blumen H.M., Buchman A.S. (2023). Adapting the reserve and resilience framework for motor and other aging phenotypes. Neurobiol. Aging.

[3] Holtzer R., Verghese J., Xue X., Lipton R.B. (2006). Cognitive processes related to gait velocity: Results from the Einstein Aging Study. Neuropsychology.

[4] Sunderaraman P., Maidan I., Kozlovski T., Apa Z., Mirelman A., Hausdorff J.M., Stern Y. (2019). Differential associations between distinct components of cognitive function and mobility: Implications for understanding aging, turning and dual-task walking. Front. Aging Neurosci..

[5] Baltes P.B., Lindenberger U. (1997). Emergence of a powerful connection between sensory and cognitive functions across the adult life span: A new window to the study of cognitive aging?. Psychol. Aging.

[6] Blumen H.M., Holtzer R., Brown L.L., Gazes Y., Verghese J. (2014). Behavioral and neural correlates of imagined walking and walking-while-talking in the elderly. Hum. Brain Mapp..

[7] Tripathi S., Verghese J., Blumen H.M. (2019). Gray matter volume covariance networks associated with dual-task cost during walking-while-talking. Hum. Brain Mapp..

[8] Montero-Odasso M., Verghese J., Beauchet O., Hausdorff J.M. (2012). Gait and cognition: A complementary approach to understanding brain function and the risk of falling. J. Am. Geriatr. Soc..

[9] Buracchio T., Dodge H.H., Howieson D., Wasserman D., Kaye J. (2010). The trajectory of gait speed preceding mild cognitive impairment. Arch. Neurol..

[10] Morris R., Lord S., Bunce J., Burn D., Rochester L. (2016). Gait and cognition: Mapping the global and discrete relationships in ageing and neurodegenerative disease. Neurosci. Biobehav. Rev..

[11] Verghese J., Robbins M., Holtzer R., Zimmerman M., Wang C., Xue X., Lipton R.B. (2008). Gait dysfunction in mild cognitive impairment syndromes. J. Am. Geriatr. Soc..

[12] Skillbäck T., Blennow K., Zetterberg H., Skoog J., Rydén L., Wetterberg H., Guo X., Sacuiu S., Mielke M.M., Zettergren A. (2022). Slowing gait speed precedes cognitive decline by several years. Alzheimer’s Dement..

[13] Ayan C., Cancela J.M., Gutiérrez A., Prieto I. (2013). Influence of the cognitive impairment level on the performance of the Timed “Up & Go” Test (TUG) in elderly institutionalized people. Arch. Gerontol. Geriatr..

[14] de Oliveira Silva F., Ferreira J.V., Placido J., Chagas D., Praxedes J., Guimaraes C., Batista L.A., Marinho V., Laks J., Deslandes A.C. (2019). Stages of mild cognitive impairment and Alzheimer’s disease can be differentiated by declines in timed up and go test: A systematic review and meta-analysis. Arch. Gerontol. Geriatr..

[15] Ibrahim A., Singh D.K.A., Shahar S. (2017). ‘Timed Up and Go’test: Age, gender and cognitive impairment stratified normative values of older adults. PLoS ONE.

[16] Párraga-Montilla J.A., Pozuelo-Carrascosa D.P., Carmona-Torres J.M., Laredo-Aguilera J.A., Cobo-Cuenca A.I., Latorre-Román P.Á. (2021). Gait performance as an indicator of cognitive deficit in older people. Int. J. Environ. Res. Public Health.

[17] Clouston S.A., Brewster P., Kuh D., Richards M., Cooper R., Hardy R., Rubin M.S., Hofer S.M. (2013). The dynamic relationship between physical function and cognition in longitudinal aging cohorts. Epidemiol. Rev..

[18] Verghese J., Wang C., Lipton R.B., Holtzer R., Xue X. (2007). Quantitative gait dysfunction and risk of cognitive decline and dementia. J. Neurol. Neurosurg. Psychiatry.

[19] Benedet M.J., Alejandre M.A. (1998). Test de Aprendizaje Verbal España-Complutense.

[20] Kaplan E.F., Goodglas H., Waintraub S. (1983). The Boston Naming Test.

[21] Williams B.W., Mack W., Henderson V.W. (1989). Boston naming test in Alzheimer’s disease. Neuropsychologia.

[22] Reitan R.M., Wolfson D. (1993). The Halstead–Reitan neuropsychological test battery. Theory and Clinical Interpretation.

[23] Facal D., Juncos-Rabadán O., Pereiro A.X., Lojo-Seoane C. (2014). Working memory span in mild cognitive impairment. Influence of processing speed and cognitive reserve. Int. Psychogeriatr..

[24] Carral J.M.C., Ayán C., Sturzinger L., Gonzalez G. (2019). Relationships between body mass index and static and dynamic balance in active and inactive older adults. J. Geriatr. Phys. Ther..

[25] da Silva L.P.G., de Araújo M.D.G.R., dos Santos Costa A., de Sousa Pedrosa B.C., da SIlva K.K.D., dos Santos T.M. (2020). Inertial sensor and Timed Up and Go test in elderly women with bone demineralization: A reliability and agreement study. Rev. Bras. Atividade Fís. Saúde.

[26] Posada-Ordax J., Cosin-Matamoros J., Losa-Iglesias M.E., Becerro-de-Bengoa-Vallejo R., Esteban-Gonzalo L., Martin-Villa C., Calvo-Lobo C., Rodriguez-Sanz D. (2021). Accuracy and repeatability of spatiotemporal gait parameters measured with an inertial measurement unit. J. Clin. Med..

[27] Weiss A., Mirelman A., Giladi N., Barnes L., Bennett D., Buchman A., Hausdorff J.M. (2016). Transition between TUG turn to sit subtasks: Is timing everything?. J. Am. Med. Dir. Assoc..

[28] Alley D.E., Shardell M.D., Peters K.W., McLean R.R., Dam T.T.L., Kenny A.M., Fragala M.S., Harris T.B., Kiel D.P., Guralnik J.M. (2014). Grip strength cutpoints for the identification of clinically relevant weakness. J. Gerontol. Ser. A Biomed. Sci. Med. Sci..

[29] Ruiz Comellas A., Pera G., Baena Díez J.M., Mundet Tudurí X., Alzamora Sas T., Elosua R., Torán Monserrat P., Heras A., Forés Raurell R., Fusté Gamisans M. (2012). Validación de una versión reducida en español del cuestionario de actividad física en el tiempo libre de Minnesota (VREM) [Validation of a Spanish Short Version of the Minnesota Leisure Time Physical Activity Questionnaire (VREM)]. Rev. Esp. Salud Pública.

[30] Asperholm M., Högman N., Rafi J., Herlitz A. (2019). What did you do yesterday? A meta-analysis of sex differences in episodic memory. Psychol. Bull..

[31] Nonnekes J., Post E., Imbalzano G., Bloem B.R. (2025). Gait changes with aging: An early warning sign for underlying pathology. J. Neurol..

[32] Wu T., Hallett M. (2005). The influence of normal human ageing on automatic movements. J. Physiol..

[33] Giovannoli J., Martella D., Casagrande M. (2023). Executive functioning during verbal fluency tasks in bilinguals: A systematic review. Int. J. Lang. Commun. Disord..

[34] Whiteside D.M., Kealey T., Semla M., Luu H., Rice L., Basso M.R., Roper B. (2016). Verbal fluency: Language or executive function measure?. Appl. Neuropsychol. Adult.

[35] Brandt T., Schautzer F., Hamilton D.A., Brüning R., Markowitsch H.J., Kalla R., Darlington C., Smith P., Strupp M. (2005). Vestibular loss causes hippocampal atrophy and impaired spatial memory in humans. Brain.

[36] Maguire E.A., Burke T., Phillips J., Staunton H. (1996). Topographical disorientation following unilateral temporal lobe lesions in humans. Neuropsychologia.

[37] Maguire E.A., Gadian D.G., Johnsrude I.S., Good C.D., Ashburner J., Frackowiak R.S., Frith C.D. (2000). Navigation-related structural change in the hippocampi of taxi drivers. Proc. Natl. Acad. Sci. USA.

[38] Griesbauer E.M., Manley E., Wiener J.M., Spiers H.J. (2022). London taxi drivers: A review of neurocognitive studies and an exploration of how they build their cognitive map of London. Hippocampus.

[39] Sternäng O., Reynolds C.A., Finkel D., Ernsth-Bravell M., Pedersen N.L., Dahl Aslan A.K. (2016). Grip strength and cognitive abilities: Associations in old age. J. Gerontol. Ser. B Psychol. Sci. Soc. Sci..

[40] Sui S.X., Hendy A.M., Teo W.P., Moran J.T., Nuzum N.D., Pasco J.A. (2022). A review of the measurement of the neurology of gait in cognitive dysfunction or dementia, focusing on the application of fNIRS during dual-task gait assessment. Brain Sci..

[41] Latino F., Tafuri F. (2024). Physical activity and cognitive functioning. Medicina.

